# Reply to the commentary “Liquid gold: do we need to fraction fresh colostrum for oral immunotherapy in premature infants?”

**DOI:** 10.1186/s13006-022-00473-w

**Published:** 2022-04-06

**Authors:** Sina Kazemian, Minoo Fallahi, Mohammad Kazemian

**Affiliations:** Neonatal Health Research Center, Research Institute for Children’s Health, Mofid Children’s Hospital, Shahid Beheshti University of Medical Sciences, Shariati Ave, Tehran, 15468- 15514 Iran

We would like to thank Pillai et al. for their thoughtful comments and appreciate their interest in our study [1]. We welcome this opportunity to respond to the comments raised in their letter. Regarding the query about the administration of colostrum in its natural form, we would like to highlight the fact that despite the strong recommendation on early feeding and minimal enteral nutrition in the first few days of life, unfortunately, some preterm infants (especially very low birth weight infants) are deprived of this optimum source of nutrition for several days or weeks after birth due to limitations like respiratory distress, poor sucking, low gastrointestinal motility, lack of digestive enzymes, etc. [2, 3]. During several experiments and centrifugation of fresh breast milk samples with the mentioned protocol, we observed many different progenitor cell groups surrounded by fatty chain cells at the bottom of the sample tube (the cellular layer). In this study, we decided to evaluate the administration of these cellular chains even before minimal enteral feeding in neonates with birthweight ≤1800 g. We tried to facilitate absorption by separating fat and proteins from the samples, and the tolerance was increased by using the minimum amount (0.1–0.2 ml) of semi-solid breast milk cell fractions (BMCFs).

We share the author’s concern regarding the lack of availability of mothers’ own milk during the first few hours after delivery. We agree and consider the lack of availability or conditions that contraindicated breastmilk feeding as the primary challenges for using this method. However, this method can encourage mothers to breastfeed by reducing anxiety and increasing the baby’s hope of survival. In addition, exclusive use of their own mothers’ milk can prevent unwanted side effects from using donor breast milk [4].

In relation to information bias, the blinding was not possible since the control group did not receive any intervention, and breastfeeding was started as soon as tolerated in both groups. We believe lack of double-blinding may have influenced the study results, as we have pointed out this as a study limitation in our discussion section [1]. The suggestion by Pillai et al. to give colostrum directly to the control group in the same manner as the intervention could be used as a solution to do blinding in future studies. To prevent selection bias, we used a computer-generated block randomization sequence. Also, the allocation concealment was done by calling in by phone and finding out what the next patient was assigned to. We have to emphasize that both participants and medical staff were unaware of the randomization list.

Regarding other factors that could impact necrotizing enterocolitis (NEC) incidence, we must state that no infant received probiotics or donor human milk in our study since these treatment strategies are not officially used in our center. But recently, the use of donated milk has become more prevalent in some other centers across the country. Since it is the first study using this novel method, we used the study results from Stoll et al. [5] to calculate the sample size. However, we believe that the low sample size may have influenced the study results, and this study is just the beginning of using this novel method. Further multi-central studies with larger sample sizes are warranted to confirm these findings.

Lastly, we observed a significantly lower in-hospital mortality in neonates with a birthweight of ≤1800 g who received BMCFs (odds ratio (OR): 0.34). Also, the difference was more significant in infants with a birthweight of < 1500 g (OR:0.24). Despite the decreasing trend for some major morbidities such as positive blood culture and NEC, we think the small number of samples and chance error may have caused *P*-value to be insignificant. In addition, early deaths in the control group could be the main reason for the underestimation of bronchopulmonary dysplasia.

We hope that this additional information clarifies the questions raised to some extent. In this study, we have tried to introduce a novel method with minimal intervention that allows us to deliver BMCFs to very low birthweight infants who may not tolerate even low amounts of early feeding. We are looking forward to developing and evaluating this method in larger studies with more frequent use of BMCFs.

## Data Availability

Not applicable.
